# Infant Eye Gaze While Viewing Dynamic Faces

**DOI:** 10.3390/brainsci11020231

**Published:** 2021-02-12

**Authors:** Lisa M. Oakes, Michaela C. DeBolt, Aaron G. Beckner, Annika T. Voss, Lisa M. Cantrell

**Affiliations:** 1 Department of Psychology, University of California, Davis, CA 95616, USA; mdebolt@ucdavis.edu (M.C.D); agbeckner@ucdavis.edu (A.G.B.); 2 Center for Mind and Brain, University of California, Davis, CA 95618, USA; atvoss@ucdavis.edu; 3 Department of Child and Adolescent Development, California State University, Sacramento, CA 95819, USA; lisa.cantrell@csus.edu

**Keywords:** infancy, eye movements, eye tracking, face processing, face race

## Abstract

Research using eye tracking methods has revealed that when viewing faces, between 6 to 10 months of age, infants begin to shift visual attention from the eye region to the mouth region. Moreover, this shift varies with stimulus characteristics and infants’ experience with faces and languages. The current study examined the eye movements of a racially diverse sample of 98 infants between 7.5 and 10.5 months of age as they viewed movies of White and Asian American women reciting a nursery rhyme (the auditory component of the movies was replaced with music to eliminate the influence of the speech on infants’ looking behavior). Using an analytic approach inspired by the multiverse analysis approach, several measures from infants’ eye gaze were examined to identify patterns that were robust across different analyses. Although in general infants preferred the lower regions of the faces, i.e., the region containing the mouth, this preference depended on the stimulus characteristics and was stronger for infants whose typical experience included faces of more races and for infants who were exposed to multiple languages. These results show how we can leverage the richness of eye tracking data with infants to add to our understanding of the factors that influence infants’ visual exploration of faces.

## 1. Introduction

Infants’ face processing has been the focus of research for decades. Since Fantz’s [1] observation that newborn infants prefer face-like stimuli to other patterns, researchers have asked how they perceive, process, and remember faces. Research has revealed that by 5 to 6 months of age, infants can remember and discriminate upright oriented faces [2] and selectively attend to faces in cluttered visual arrays [3]. The availability of eye tracking systems that can be used with young infants has allowed us to also probe how infants visually investigate faces, presumably providing understanding into how they learn about faces.

One of the most robust findings from studies of infant eye gaze during face viewing is that infants tend to fixate on the eyes and mouths of faces [4,5,6]. Oakes and Ellis [4] presented static images of racially diverse faces to a group of racially diverse infants between 4 and 12 months of age. In general, infants were biased to look at the eyes, but across the ages, infants began to look at regions in the lower half of the face, suggesting a shift from being eye focused to scanning faces more broadly, and particularly an increased attention to the mouth. An even stronger bias to look at mouths has been observed in dynamic stimuli. Xiao et al. [5] found that Chinese 3- to 9-month-old infants looked more at the mouths of dynamic Chinese faces (e.g., women chewing with a neutral expression) than at static images of those same faces. Ayneto and Sebastian-Galles [7] found that monolingual 8-month-old infants looked longer at the mouth (than the eyes) of a White woman engaged in dynamic, non-linguistic activities (e.g., laughing).

However, this effect is not universal. In a mostly White sample of 3- and 9-month-old infants, Wilcox et al. [6] reported that older infants actually showed a stronger preference to look at the eyes of a dynamic talking White woman’s face compared to younger infants, who had a stronger preference for the mouth region. Such discrepancies are especially problematic because infants’ looking to the eyes and mouth have been used as an indicator of “typical” development, as well as a way of identifying children at risk for disorders such as autism [8]. Thus, it is important to understand more about the factors that determine infants’ biases to look at the upper or lower regions of faces.

Even in adults, relative interest in the eyes and mouths of faces varies. Wegner-Clemens et al. [9], for example, found individual differences in adults’ preference for the eyes or mouth when viewing human faces, and that preference for eyes or mouth varied as a function of task. Specifically, when adults were viewing a speaking face and their task was to identify the speech, they showed more preference for the mouth region than when looking at non-moving faces or when making non-language related judgments about moving faces. These preferences also vary as a function of the specific faces being viewed. In a face memory task, for example, Chinese adults looked longer at the eyes of other-race White faces than at own-race Chinese faces; they fixated on the nose and mouth of own-race Chinese faces more than the other-race White faces [10]. European adults also preferred the eyes of White faces and the lower regions of Asian faces [11], raising the possibility that these effects reflect characteristics of different faces.

Infants’ biases to look at the eyes or mouth seem to be related to differences in their experience and developmental level. For example, selective attention to the mouth is thought to be related to infants’ language development [12]. Lewkowicz and Hansen-Tift argue that as infants begin to process language, they focus their attention to regions of the face important for that processing. Indeed, Tenenbaum et al. [13] observed that the level of mouth bias at 12 months of age predicted children’s vocabulary several months later. In support of the general proposal that infants’ looking at the mouth is related to their language development, infants look more at mouths—and this preference extends later into infancy—when viewing someone speaking a non-native language than when viewing someone speaking their native language [12,14]. Although not precisely the same effect, Berdasco-Muñoz et al. [15] found that 6- and 8-month-old healthy full-term infants had stronger preference for the eye region (relative to the mouth region) when viewing a woman reading a children’s story in their native French than when viewing her read the story in unfamiliar English. These infants did not show a preference for the mouth (relative to the eyes) even when hearing non-native language, but they did show stronger attention to the mouth relative to the eyes when the language was unfamiliar. Together, these results seem to indicate that when language processing is especially difficult, i.e., when the language being spoken is unfamiliar, infants are even more drawn to the mouth region. This is generally consistent with the notion that infants’ looking is related to their learning; they look at regions that are useful or informative when processing a visual stimulus (e.g., see [16,17]). Thus, one factor that seems to influence infants’ looking to the mouth region (relative to their looking at the eye region) is their exposure to language and their developing language ability.

Not all studies have observed a bias for mouths, however. As described above, Berdasco-Muñoz et al. [15] found an overall preference for eyes, but the relative difference between the eyes and mouth was influenced by the language they were learning. Smith et al. [18] observed that 5- to 8-month-old infants showed a preference for the eye region, and their relative looking to the eyes and mouth did not vary with the kind of speech (infant directed or adult directed). Overall, these findings indicate that language processing and exposure may moderate infants’ looking to the mouth relative to the eyes, but it is not clear when (or whether) a preference for the mouth over the eyes should be observed.

The bias to look at the eyes or mouth may also be related to how familiar infants are with the race of the face being viewed. White infants tend to look more at the eyes of their own-race faces and the mouths of other-race faces, at least by 9 months of age. This pattern also might reflect infants’ attention to the mouth when exposed to more unfamiliar or difficult to process faces. For example, White infants showed a bias to prefer eyes of White own-race faces and to prefer mouths of other race Asian faces [19] or African faces [20,21]. Once again, however, this effect is not universal. Two studies (using the same stimuli) failed to show differences in how Chinese or White infants looked at the eyes or mouths of own- and other-race faces [22,23]. Moreover, Geangu et al. [24] found that White 7-month-old infants in the UK showed a stronger preference for the mouths of both own (White) and other (Asian) race faces, whereas Asian 7-month-old infants in Japan showed a stronger preference for the eyes for both own (Asian) and other (White) faces, suggesting that culture, and not face race, contributes to differences in where infants look when viewing faces.

In both the domains of language experience and face experience, there is an indication that the diversity of infants’ experience may be important. The mouth preference is even more pronounced for bilingual infants [14], even when viewing non-linguistic stimuli [7]. This may seem to contradict the conclusion that infants are more likely to look at the mouths of faces when processing is more difficult. However, it may indicate that bilingual infants are better in general at using the mouth region to disambiguate or process a speaking face, perhaps because of their more diverse language experience. Diversity of face experience also is related to how infants scan faces. Ellis et al. [25] found that White 8-month-old infants from a racially homogenous community (Grinnell, Iowa) looked longer at the eyes of other-race Asian American faces than at the eyes of own-race White faces. White 8-month-old infants from a racially heterogeneous community (Sacramento Valley, California) did not distribute their looking differently to the eyes and mouths of own- and other-race faces. Gaither et al. [26] observed that when White, Asian American, and biracial (White/Asian American) 3-month-old infants were habituated to White and Asian American faces, the biracial infants showed a different pattern of scanning the upper and lower regions of the faces than either the White or Asian American infants. Similarly, Tham et al. [27] found that Malaysian-Chinese infants, who experience racial diversity in their daily lives, did not show the same development of the “other-race” effect as infants raised in a single-race environment. This limited literature, therefore, suggests a more diverse experience—either in their exposure to language or in their exposure to faces of different races—contributes to infants’ fixation on of eyes and mouths as they view faces.

Thus, taken as a whole, the findings from this literature suggest that infants’ learn how to look from their experience, consistent with other findings showing a more direct connection between experience and infants’ visual inspection (e.g., how infants with and without pets look at images of dogs and cats [16]). Moreover, Kovack-Lesh et al. [17] suggested that infants’ experience may contribute to their attentional strategy when looking at relatively familiar and unfamiliar visual stimuli. In the case of infants’ visual inspection of faces, experience both learning about faces from looking at faces and learning language from watching (and listening to) speaking faces may contribute to the attentional strategies infants adopt when they are presented with new face stimuli.

In the current study, we recorded eye gaze in infants between 7.5 and 10.5 months of age as they viewed White and Asian American women reciting a nursery rhyme (vocalizations removed and replaced with music) in either infant-directed or adult-directed speech (i.e., as described later, they were instructed to recite the nursery rhyme as if they were speaking to an infant or speaking to an adult). One of our goals was to understand how infants’ eye gaze varied as a function of these stimulus characteristics (face race and speech type). This study differed from previous studies in several important ways. First, unlike most studies examining the effect of race on infants’ looking behavior, our stimuli were dynamic. Although studies from Lee and colleagues have examined infants’ scanning of own- and other-race faces using dynamic stimuli [20,21,22,23], others have asked this question using static stimuli [4,19,24,25]. Moreover, the dynamic nature of our stimuli (e.g., a woman reciting a nursey rhyme) was more typical of the stimuli assessing the effect of language development and experience on infants’ visual inspection faces [7,12,14]. Thus, this aspect of our stimuli allows us to bridge these two literatures.

Second, we asked how infants’ visual inspection of the faces varied as a function of their experience. However, rather than examine a single aspect of experience, we evaluated three different features of infants’ experience. One measure of experience was their mother’s race. A second measure was derived from parent report of the infants’ exposure to and experience with faces of different races. In addition, because we routinely ask parents about their infants’ language experience, we also had information in this sample about whether they had monolingual or multilingual experience. Thus, the current study is different from previous studies because we analyzed their eye gaze considering three different aspects of their experience.

Finally, in the current study, we did not identify a single dependent variable and a single analysis to present. Many studies examining infants’ eye gaze while viewing faces focus on their overall looking times to regions such as the eyes and mouth [4,6,18,23]. Others have examined the patterns of locations of individual fixations [20], and increasingly researchers are reporting moment-to-moment time courses to understand how eye gaze unfolds over time [28,29]. Thus, it is not clear a priori how the data should be analyzed. A significant problem in psychological science is that there are many different variables to analyze and many ways to analyze any set of data [30,31]. This is what Gelman and Loken [30] refer to as “the garden of forking paths”. Recently, the multiverse analysis approach [32] has been suggested as one solution to this problem. For example, by varying the definitions or criteria for the levels of the independent variables, researchers can conduct the same (or similar) analyses on different subsamples. Others have conducted analyses on different variables or indices of the construct of interest [33]. Conclusions are drawn by examining what effects are consistent across these variations, thus minimizing the likelihood that the significant effects reported reflect specific decisions made by the researchers.

Here, we adopt a similar approach and report different analyses on a single dataset. Our approach was to examine infants’ looking at different time scales. At the coarsest time scales, we aggregated infants’ looking across trials and conducted analyses on infants’ looking as a function of stimulus type, which is the typical approach adopted in the literature. In addition, we used linear mixed-effects models to analyze looking at finer time scales, first at the level of trial (summing over fixations within a trial) and then at the level of individual fixation. Finally, we used a time course analysis to evaluate how infants’ looking changes moment by moment within a trial. As in a multiverse analysis approach, our conclusions are based on those effects that are robust and consistent across many different dependent variables and analyses.

## 2. Materials and Methods

### 2.1. Participants

We included 98 infants between 7.5 and 10.5 months of age (*M* = 273 days, *SD* = 31, range 217–320) in our final sample. There were 46 boys and 52 girls. In our final sample, we included only infants born at term (e.g., no more than 21 days before their due date), with no history of neurological or chronic illness, no birth complication (e.g., no time in the NICU), no familial risk of colorblindness, and no reported vision or hearing problems.

Parents reported race and ethnicity separately, as required by the National Institutes of Health. In our sample, parents reported that 43 were White, 7 were Black or African American, 13 were Asian American, 27 were mixed race, and 8 did not report this information. In addition, parents reported that 32 infants were Hispanic or Latino; of these infants, 8 were White, 2 were Black or African American, 2 were Asian American, 12 were mixed race, and 8 did not report race. Parents also reported the highest level of education attained by the mother; of the 96 mothers who reported this information, 61 had earned at least a 4-year degree, 30 had completed some college or a 2-year degree, and 5 had a high school diploma. Parents also reported infants’ language exposure. Of the 95 infants for whom this information was reported, 66 infants were exposed to English more than 75% of the time, 6 infants were exposed to a language other than English more than 75% of the time, and 23 infants were exposed to multiple languages.

Infants were recruited from our database of potential infant research participants. We obtained infant names from the California State Office of Vital Records. All parents who have an address within 30 miles of our lab were sent information with instructions on how to volunteer and be included in our database of potential research participants. The information was sent in the first few months after the birth was recorded. Parents indicated their willingness to participate by replying via phone, email, a web form, or returning a postage-paid card. When infants reached the appropriate age for this study, we contacted parents who indicated an interest in participating in general about participating in this study specifically. Any parent who was interested was offered participation after an initial screening to identify infants with vision or hearing problems, serious medical or neurological problems, a family history of color blindness, or who were born premature (i.e., more than 21 days before their due date). Infants who met our eligibility criterion were scheduled for appointments.

An additional 27 infants were tested but were not included in the final sample due to experimenter error (e.g., presenting the wrong stimulus, *n* = 7), fussiness as determined by the online experimenter (*n* = 4), inability to calibrate (*n* = 14), or parental interference (*n* = 2). In addition, we tested 6 infants and then later determined that they were ineligible to participate (e.g., were premature, had health problems); their data were discarded.

### 2.2. Materials

We developed a face experience questionnaire, similar to that used by Rennels and Davis [34]. In this questionnaire, parents were asked to consider their infants’ experience over the previous week and indicate whether the infant had brief or involved experiences with individuals of particular races, the full questionnaire can be found in the Supplementary Materials section on Infant eye gaze while viewing dynamic faces OSF project page [35] *Involved interactions* were defined as interactions in which the person was less than 5 feet away from the infant and in the infant’s view for more than 5 min. *Brief interactions* were defined as interactions in which the other person was over 5 feet away from the infant and in the infant’s view for less than 5 min. Parents were asked to indicate how many brief or involved interactions infants had with Asian women, White women, and Black women (parents also rated infants’ experience with men, but for this study we only considered the responses with respect to interactions with women because our stimuli were all women’s faces). Parents also reported whether the level of interaction was typical for their infant.

In addition, parents completed a general demographics questionnaire in which they reported their infant’s race, race of the mother, education of the mother, and any infant health problems. Parents also reported whether the infant had any regular caregiver, and if so, they reported the gender, approximate age, and race of this caregiver. On this questionnaire, parents were asked questions about their infants’ language experience. Parents reported (1) the primary language spoken in the home, (2) whether the infant regularly heard any other languages, and if so (3) what other languages did the infant hear, and (4) what proportion of the time infants heard each language. We used this information to classify infants as English monolingual or multilingual as described later in Section 2.6.

**Figure 1:**
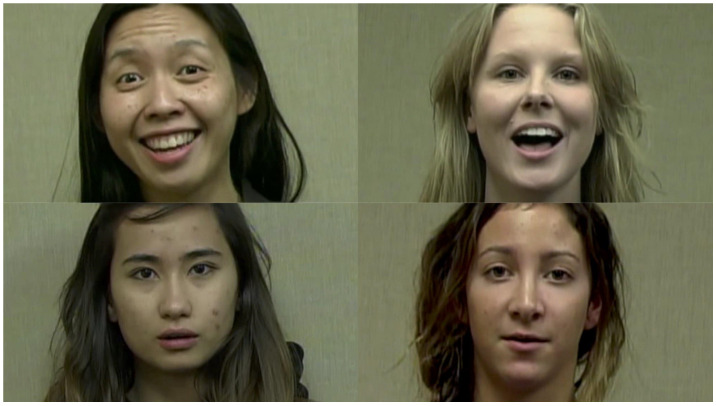
Screenshots from four of the stimulus movies used in this study. On the top row, the women are using infant-directed speech, and on the bottom row the women are using adult-directed speech. The women on the left are Asian American, and the women on the right are White.

Our primary experimental stimuli were a series of 8 movies, 7.5 to 10.5 s in duration. There were four models used to create these movies. Two women self-identified as White and two women self-identified as Asian American (see Figure 1). Each movie was a view of one woman’s head and shoulders as she recited “Humpty Dumpty”. Each woman recited the nursery rhyme in two movies. For the *infant-directed* movie, we asked women to recite the rhyme as they would to an infant, using the facial expression and tone of voice that they would use in that context. For the *adult-directed* movie, we asked women to recite the nursery rhyme neutrally, as if to an adult in a more professional setting. Thus, as a result, the women were more animated and showed more emotions in the infant-directed movies than in the adult-directed movies.

We removed the audio and replaced it with classical music, so the infant could not hear what the woman was saying. The same music was played on each trial (Debussy’s “Maid with the Flaxen Hair” performed by Kirk Trevor, Richard Stoltzman, and the Slovak Radio Symphony Orchestra). In this way, there were no auditory cues differentiating type of speech, race of the model, or the particular stimulus, and the music would not lead to any specific or systematic gaze preferences. We used music because this is a common procedure to maintain infants’ interest in the task in general [4,36,37]. This allowed us to examine the specific effects of the face movement while controlling for any influence of the auditory component of language. Previous research has shown that infants’ preference for infant-directed speech can be obtained even when the stimuli are silent [38], and the visual cues may be more compelling to infants than vocal cues [39]. The 8 stimulus movies can be found in the Supplementary Materials section on Infant eye gaze while viewing dynamic faces OSF project page [35].

### 2.3. Apparatus

Eye movements were recorded using an SMI-RED M eye tracker (SensoMotoric Instruments, Teltow, Germany), which captured eye gaze at a rate of 120 Hz. The eye tracker was attached to the bottom of a 22-in LCD monitor (1680 × 1050 resolution). A web camera attached to the top of the monitor captured the infants’ head and body position throughout the duration of the experiment. The monitor was affixed to an ergo arm that allowed the experimenter to position it to optimally locate each infant’s eyes in the center of the detection radius of the eye tracking system. A Dell laptop supplied by SMI was used to monitor the participant and run the experiment.

### 2.4. Procedure

Infants were seated on a parent’s lap, approximately 60 cm from the stimulus presentation monitor and the eye tracker. Parents were seated in a stationary chair and wore a pair of opaque glasses that obstructed their view of the stimuli during the session (and ensured the eye tracker did not detect the parent’s eyes). A curtain separated the infant from the experimenter and the equipment.

We used the SMI software Experiment Center (SensoMotoric Instruments, Teltow, Germany) to present the experiment. Each session began with a 5-point calibration procedure in which a looming circle was presented in the upper left, upper right, lower left, lower right, and center locations. When the infant looked at each of the calibration points the eye tracking system calculated the infants’ point of gaze (POG) using the corneal and pupil information captured by the eye camera. A verification procedure was initiated immediately following calibration; infants’ POG was checked by presenting an attention grabbing, animated yellow duck that shook and made noise at the five locations. If this verification revealed poor calibration, the experimenter reinitiated the calibration procedure.

The experimental phase began immediately after calibration. Each infant was presented up to 8 trials (two trials of each stimulus type). Before each trial, there was a blinking fixation cross accompanied by a bell ringing that alternated with a colorful spinning toy. This location of the fixation cross roughly corresponded to the upper bridge of the nose of the stimulus face. When the SMI detected that the infant fixated this attention-getter for 200 ms, an experimental trial began. The trials were presented in blocks of four, with one trial of each type (Asian American infant directed, Asian American adult directed, White infant directed, and White adult directed) in each block. Thus, if infants saw all 8 trials, they saw each of the four trial types twice. Between blocks, we presented a short clip from a children’s video to maintain infants’ attention to the task in general. If all 8 trials were presented, the session was approximately 1 ½ to 2 min in duration.

### 2.5. Data Processing

**Figure 2:**
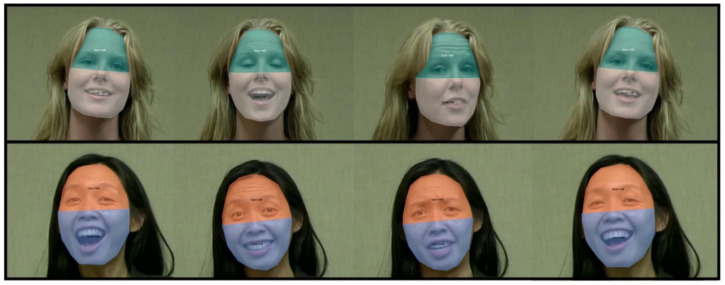
Examples of our dynamic AOIs for two of our stimuli. Note that the AOIs move with the face, so they capture the same region of the face even as the face moves.

The data recorded by the SMI eye tracking system were initially processed in and exported from SMI’s software BeGaze. We used BeGaze to create dynamic AOIs for the upper and lower halves of the faces. We created AOIs that bisected the face and were approximately 8.5 cm high by approximately 16 cm wide (8˚ by × 15˚ visual angle at a viewing distance of 60 cm). As the face moved, the AOIs also moved; thus, the AOIs were relative to the face and not to specific locations on the screen (see Figure 2). That is, they were dynamic and moved with the stimuli. As such, a single fixation to the same location on the screen could be labelled as a fixation to the upper half or the lower half of the face, depending on where the face stimulus was located at the moment of that fixation. Note that the AOI for the upper half included the eyes, forehead, and nose bridge, whereas the AOI for the lower half included the mouth, the tip of the nose, and the nostrils.

We extracted two measures from BeGaze. First, we filtered the data in BeGaze into fixations using the standard fixation parameters for low-speed (<200 Hz) eye tracking. Fixations were defined as any period of gaze that was at least 80 ms in duration, with maximum dispersion of 100 px. BeGaze produced a file that included for each fixation the duration of the fixation, the stimulus, trial, fixation index (e.g., which fixation it was in the trial), and the AOI (upper or lower half) that the fixation fell in. The second measure was the XY coordinates of the eye gaze at each individual sample (e.g., 120 data points per second). BeGaze produced a file that included for each sample: whether the eye gaze was in the upper or lower half of the face (or neither), the stimulus, and trial.

We imported these data files into R and combined them with infant demographics. In R we applied our exclusion criteria. To be included in our analyses, we required that there be at least 1000 ms of recorded looking total on a trial. Of the initial 728 trials, 38 trials did not meet this criterion and were excluded. One additional trial from one infant’s session was dropped because it was cut short by the experimenter. Because many of our analyses were mixed-effects, multilevel models that would adjust for the number of trials completed, we included in our final dataset any infant who had at least one trial that met this criterion.

We used these data to derive measures that reflected the sum of looking across all fixations in a trial, generating the total looking to the upper and lower regions of the face in each trial. From the samples data, we created bins of 250 ms to evaluate how infants’ preference for the lower half changed over the course of the trials. For each bin of 250 ms, we calculated the proportion of samples that were in the lower half by dividing the number of samples in the lower half by the total number of samples in that period that were in either the upper or lower half. All analyses were conducted on the same 689 trials that met our inclusion criteria.

### 2.6. Deriving Experience Variables

For our analyses, we identified three separate experience variables. First, we used a parental report of maternal race to generate a *maternal race* variable. Because our goal was to determine whether infants’ eye gaze differed for relatively familiar (own) race and relatively unfamiliar (other) race, we classified maternal race into *White*, *Asian American*, or *Other*. Mothers who were reported to be White were classified as White, regardless of whether or not they also identified as Hispanic. Mothers who were Asian American or Asian American and White were classified as Asian American. Mothers were reported to be some race other than Asian American or White, or who did not report race but were reported to be Hispanic were classified as Other. This yielded 48 infants with White mothers, 22 infants with Asian American mothers, 28 infants with non-White/non-Asian American mothers (see Table 1).

Next, we used parent reports of infants’ experience with faces as described earlier to classify each infants’ experience as relatively *high*, *medium*, or *low*, with respect to racial diversity of the women’s faces they typically encounter. This diversity was determined by considering the race of the mother, the race of any significant female caregiver, and parental responses to our face experience questionnaire. We identified from the face experience questionnaire whether infants typically had exposure to Asian American, White, and Black faces. Although our stimuli were Asian American and White women, for understanding diversity we included infants’ exposure to Black women’s faces as well. Thus, this index does not reveal how much exposure infants have to Asian American or White faces, but rather their exposure to many different types of faces. We classified infants who had experience with three racial groups as high (recall that we classified mothers who were Hispanic and did not report a race as “Other”). For example, an infant with a Hispanic/Non-White mother who was reported to typically interact with at least one Asian American woman and at least one White woman would be categorized as having relatively high diversity in their face experience. We classified infants who had experience with two racial groups as medium. For example, an infant who had a White mother and an Asian American caregiver, but no experience with Black women, would be categorized as having relatively moderate diversity in their face experience. Finally, we classified infants who had experience with only one racial group as low. For example, an infant who had an Asian American mother and an Asian American caregiver and no experience with White or Black women would be categorized as having relatively low diversity in their face experience. We classified the diversity of the experience for 89 infants (9 parents did not complete our face experience questionnaire). Of these infants, 19 were high, 39 were medium, and 31 were low (see Table 1).

Finally, we used parent reports of infants’ language experience to classify infants’ language experience. We identified 66 infants who were monolingual English; these infants were exposed to English more than 75% of the time. We identified 23 multilingual infants; these infants were exposed to multiple languages, and no one language more than 75% of the time. The remaining infants were monolingual for a language other than English (*n* = 6) or their language exposure was not reported (*n* = 3).

As seen in Table 1, there was little overlap in how infants’ experience was categorized. Across all mother’s race groups, infants had high, medium, or low diversity of experience. Similarly, infants in each mother’s race and diversity group were monolingual or multilingual. Thus, our analyses of each of these three experience variables will reflect different groupings of infants.

### 2.7. Analysis Plan

As described earlier, we did not have a single set of analyses we planned to evaluate infants’ looking data as they viewed these stimuli. Instead, we explored the effects of different types of experience on infants’ looking behavior by adopting an analytic approach like the multiverse analysis approach [32]. Two aspects of our study were varied in our analyses. First, because we did not determine a priori the best index of experience on infants’ face processing, we present here exploratory analyses on multiple indices of experience to probe for robust and consistent effects (see [33] for another example of this general approach). Specifically, we present separate analyses of experience as indexed by mother’s race, diversity of race experience, and language experience. Note that this is not precisely the same approach as multiverse analyses in which different groupings are explored by varying inclusion criteria [32]. Rather, here we are asking how different indices of experience that reflect questions asked in the literature relate to infants’ gaze will looking at faces. Comparing these different indices in the same sample will allow us to establish whether experience in general is related to infants’ behavior, in which case we will see similar effects across different indices of experience, and how specific aspects of experience are related to infants’ looking towards the upper and lower regions of our face stimuli.

Second, because the work in the infant eye tracking literature has used many different DVs, it was not obvious a priori which DV would be most appropriate here. Thus, we calculated several different DVs from our data and examined how stimulus characteristics (face race and speech type) and infants’ experience were related to each of those DVs. The approach here was more like the multiverse analytic approach, in which our goal was to identify effects that were consistent across multiple DVs. Rather than arbitrarily selecting a single DV, or “cherry picking” the DV that revealed the most interesting results, here we transparently report the analyses of several different DVs and draw conclusions about effects that are robust and observed across multiple DVs. We will be more cautious about the conclusions we draw about effects that are not revealed in analyses of multiple DVs.

First, as is common in the infant eye tracking literature, we examined infants’ responding by aggregating their responses across several trials of the same condition, and entering those scores into mixed model Analyses of Variance (ANOVAs) using the package *ez* [40]. This *condition-level* analysis will allow comparisons to other work in the field and will reveal whether infants’ overall bias to look at the lower region varies as a function of the characteristics of the stimulus or the infants’ experience. The ANOVAs included speech type as a within-subjects variable and experience as a between-subject variable. Face race was also included as a within-subject variable for any analysis including experience variables related to race (i.e., mother’s race and diversity of face race experience); experience with different races may be meaningfully related to the race of the stimulus, but it is not clear how language experience would be meaningfully related to the race of the stimulus. Thus, we did not include face race as a variable when analyzing language experience.

The ANOVA approach is limited in several ways. First, only infants who have data for all four trial types (face race crossed with speech type) can be included in these analyses. Thus, our analyses were conducted on only 82 infants of our total sample of 98 infants. Second, an ANOVA on aggregate scores has a low temporal resolution; we can draw conclusions about differences in infants’ responding on specific trial types, but not how their responding varied across or within trials. Finally, ANOVAs do not allow us to incorporate random effects (e.g., stimulus, participant) in addition to fixed effects (e.g., age, language condition) in the analysis, and thus we are not able to account for sources of variation in the data due to individual participants or specific stimuli.

To address these issues, we conducted several analyses on our other DVs using linear mixed-effects models. This approach allows us to include infants even if they do not have all four types of trials, and thus all 98 infants were included in these analyses. Because these analyses can be conducted on each trial or fixation, they have a finer temporal resolution than analyses relying on aggregate scores. As a result, they will allow conclusions about variations in infants’ responding across or within trial. Finally, these models include both fixed and random effects.

We used linear mixed-effects models to conduct *trial-level* analyses examining infants’ responding on each trial using the packages *lme4* [41] and *lmerTest* [42]; omnibus *F*-statistics were used to evaluate the significance of the fixed effects from these models [43]. In these analyses, the DV was the total duration of looking (summed across individual fixations) to the upper and lower regions of the face. This trial-level analysis provided insight into how infants’ preference for the lower region varied across trials, as well as controlling for variations in preference due to characteristics of individual stimuli.

We also conducted *fixation-level* analyses, evaluating each individual fixation separately to determine how infants’ biases to look at the lower half manifests at the level of individual fixations. We conducted two sets of fixation-level analyses. First, we analyzed the duration of each fixation using the approach just described. That is, each individual fixation on each trial was treated as a repeated measure, allowing us to evaluate how fixation duration changes over subsequent fixations within a trial as well as across individual trials. Second, we analyzed the *location* of each fixation using logistic mixed-effects models with the *afex* package [44], and Chi-square likelihood ratio tests to evaluate the significance of the fixed effects from the models. This is similar to the approach described for fixation duration, except that here we are assessing how the location of fixations changes over repeated measures, rather than duration. Together, these trial-level and fixation-level analyses allow us to evaluate our variables of interest across multiple nested repeated measures (e.g., trial level, fixation level) while accounting for participant- and stimulus-level variation in our measures of interest.

Our linear mixed-effects models included fixed effects of speech type (2 levels: infant directed and adult directed), race (2 levels: Asian American and White), and experience (mother’s race, diversity of face experience, or language experience), to evaluate our main variables of interest. As with the ANOVAs, we included face race only when probing the effect of race experience. The models also included a fixed effect of AOI (2 levels: upper half and lower half) to evaluate infants’ relative interest in the two halves of the face.

Our models also included a fixed effect of age (continuous value; range: 217–320) as a covariate. For all analyses, we included a random intercept for the grouping variable *participant* to account for the repeated measures (individual trials and individual fixations) nature of the data. We also include a random intercept for each *stimulus* to account for the fact that the stimuli we selected for this study represent a *random* sample from a population of all possible race–speech type pair possibilities [45,46] and to increase the generalizability of our findings [47]. Finally, these models included trial (continuous value; range: 1–8) and/or fixation index as fixed effects as appropriate (to make the logistic models converge, we scaled age, trial number, and fixation index). The models will be specified in the relevant Sections below.

We extracted marginal means to examine significant fixed effects and interactions using the *emmeans* package [48]. Post-hoc comparisons were evaluated either using Tukey’s HSD, using the emmeans package with multcomp [49], or the *False Discovery Rate* approach [50] in the *stats* package in R [51]. For our logistic mixed-effects models, probabilities were back-transformed from the logit scale to aid in interpretation and visualizations, but contrasts are performed on the original logit scale.

Finally, we conducted a *time course* analysis to examine moment-to-moment changes in infants’ looking behavior. To examine the time course, we used an approach described by Beckner et al. [29]. This approach involves calculating subject-weighted averages for each individual time bin and conducting uncorrected *t* tests on these averages. Clusters of consecutively significant *t*-tests are then identified and summed to calculate the *cluster mass* for a given set of time bins. These clusters are then evaluated against a null distribution generated from random permutations of the observed data to determine whether they are significantly different from what we would expect by chance. We randomly permuted the data 1000 times and recorded the largest cluster mass on each iteration of the permutation to generate the null distribution for each of our time course analyses.

## 3. Results

All the code and data used to generate these analyses can be found in the Supplementary Materials section on Infant eye gaze while viewing dynamic faces OSF project page [35]. Overall, infants contributed an average of seven trials (*SD* = 2; range 1 to 8 trials) to our analyses. Their average looking on each trial was 5876.70 ms (*SD* = 2623.74 ms). In the following sections, we report our analyses as described in the Section 2.7. earlier.

### 3.1. Overall Preference

**Figure 3:**
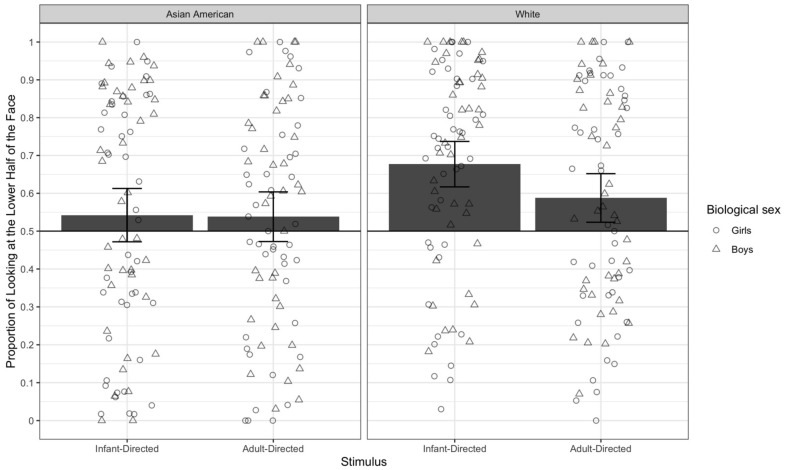
Mean preference for the lower half of the face for each stimulus type, collapsed across all infants. The line bisecting the graph at 0.50 represents chance, or equal looking at the upper and lower regions. Each open circle represents the score for an individual infant. Error bars represent 95% confidence intervals. The biological sex of each infant is indicated by the shape of the point.

The means for each of the four trial types are in Figure 3. It can be seen that, overall, infants had stronger lower half preferences for the White faces than for the Asian American faces. In addition, infants had a stronger lower half preference for White faces using infant-directed speech. These observations were confirmed by conducting three separate ANOVAs (one for each experience variable). For each ANOVA, the dependent variable was the proportion of infants’ looking to the lower half of the faces for the different trial types. Each ANOVA included one grouping independent variable, characterizing some aspect of their experience. These independent variables were *mother’s race* (White, Asian, or Non-White/Non-Asian), *diversity of race experience* (low, medium, or high), and *language experience* (monolingual or multilingual) (see Table 1).

For the two experience variables related to race (mother’s race and diversity of race), we included face race and speech type as within-subject variables, and infants contributed four scores to the analysis (proportion of looking to the lower half for Asian American/infant directed, Asian American/adult directed, White/infant directed, and White/adult directed). Thus, we examined the effect of mother’s race with a 2 (face race) by 2 (speech type) by 3 (mother’s race) ANOVA on the proportion of looking to the lower half, and we examined the effect of diversity of racial experience with a 2 (face race) by 2 (speech type) by 3 (diversity of experience) ANOVA on the proportion of looking to the lower half. For the experience variable unrelated to race (language), only speech type was included as a within-subject variable and infants contributed two scores (infant directed and adult directed, collapsed across face race). Thus, we examined the effect of language experience with a 2 (speech type) by 2 (language experience) ANOVA on the proportion of looking to the lower half. Because we did not have race diversity and/or language information on all the infants, each of the reported analyses used a different subsample of infants.

All three ANOVAs yielded significant effects of speech type: ANOVA on mother’s race, *F*(1, 79) = 4.41, *p* = 0.023, ANOVA on diversity of experience, *F*(1, 72) = 5.40, *p* = 0.023, ANOVA on language, *F*(1, 71) = 8.63, *p* = 0.004. Thus, regardless of which subset of data were included, the analyses revealed that infants spent a higher proportion of their looking to the lower halves of dynamic stimuli involving infant-directed speech than those involving adult-directed speech. Recall that because we replaced the vocalizations in our stimuli with music, this effect actually means that infants’ preferences for the lower half of faces was likely influenced by the visual features associated with infant-directed speech—more head movement and more positive affect.

The two ANOVAs including face race also revealed significant main effects of face race, ANOVA with mother’s experience, *F*(1, 79) = 32.87, *p* < 0.001, and ANOVA with diversity of experience, *F*(1, 72) = 28.39, *p* < 0.001, due to infants devoting a higher proportion of their looking to the lower halves of White faces than of Asian faces. Both of these ANOVAs also revealed a significant interaction between face race and speech type, ANOVA on mother’s race, *F*(1, 79) = 6.24, *p* = 0.015, ANOVA on diversity of experience *F*(1, 72) = 6.74, *p* = 0.011. As clear in Figure 3, infants devoted the highest proportion of looking to the lower halves of White women’s faces when they were using infant-directed speech, and the lowest proportion of looking to the lower halves of Asian American faces using adult-directed speech. This impression was confirmed with a series of *t*-tests comparing the means including all 82 infants who had data on all four test types and using Tukey’s HSD to adjust for multiple comparisons. The proportion of looking to the lower halves of faces was significantly greater when infants viewed White women using infant-directed speech than when they viewed White women using adult-directed speech, *t*(143) = 3.47, *p* (adjusted) < 0.01, Asian American women using adult-directed speech, *t*(141) = 5.23, *p* (adjusted) < 0.001, or Asian women using infant-directed speech, *t*(143) = 5.53, *p* (adjusted) < 0.001. No other post-hoc *t*-tests were significant, with a *p* (adjusted) > 0.30.

None of these ANOVAs yielded significant effects or interactions with experience. Thus, these analyses suggest that stimulus factors, but not differences in experience, are related to how infants’ scan these dynamic faces.

### 3.2. Trial-Level Analyses

Our *trial-level analyses* examined infants’ eye gaze as summarized across fixations within a trial. For these analyses we analyzed the duration of looking to the upper and lower region in each trial. We fit three separate models, each including a different experience variable as a fixed effect: model 1 included mother’s race (3 levels: Asian American, White, and Other), model 2 included diversity of race experience (3 levels: high, medium, and low), and model 3 included language experience (2 levels: English-monolingual and multilingual). Each model also included fixed effects of AOI, trial number, and age in days and random effects of participant and stimulus. Because we were interested in the effects of stimulus characteristics and infant experience on their looking behavior, we examined up to 3-way interactions between all variables except trial number and age in days; these last two variables were included in the models, but not in the tests of interactions with the other variables. The models are specified as follows (note that Stimulus Race was only included in the models including the experience variables mother’s race and race experience):

DV ~ (Experience variable + Stimulus race + Speech type + AOI ^3 + Trial number + Age in days + (... | Participant) + (... | Stimulus)

**Figure 4:**
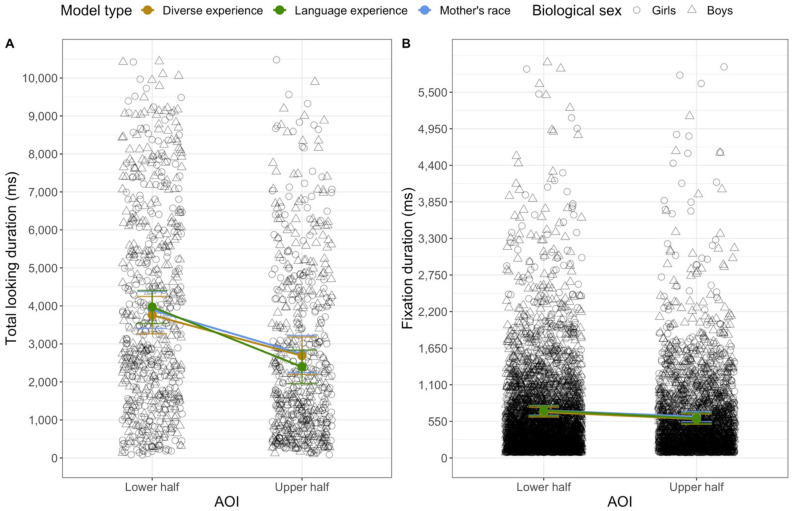
Duration of (**A**) looking summed across fixations within a trial and (**B**) for each individual fixation on each trial to the upper and lower halves, averaged across infants and trial types. Each open circle is the duration on a single trial from a single infant (**A**) or a single fixation on a single trial from a single infant (**B**). The lines represent the estimated marginal means for each of the three models, and the error bars represent 95% Sidak-adjusted confidence intervals calculated from the models. Note, for clarity, the graph for the fixation durations does not include three fixations that were between 6000 and 8000 ms, but those fixations were included in the model. The biological sex of each infant is indicated by the shape of the point.

The three models each revealed omnibus fixed effects of trial: the model with mother’s race, *F*(1, 1136.07) = 9.18, *p* = 0.003, the model with diversity of experience, *F*(1, 1034.10) = 10.79, *p* = 0.001, and the model with language experience, *F*(1, 1032) = 7.97, *p* = 0.005. These effects reflect the fact that infants’ looking decreased over trials (see Table 2). Each of the analyses revealed an omnibus fixed effect of AOI: the model with mother’s race, *F*(1, 1118.48) = 70.80, *p* < 0.001, the model with diversity of experience, *F*(1, 1031.07) = 52.92, *p* < 0.001, and the model with language experience, *F*(1, 1011.58) = 105.33, *p* < 0.001. As seen in Figure 4, averaged across trials, infants looked longer at the lower region (*M* = 3548.187 ms, *SD* = 2760.665 ms) than at the upper regions of the faces (*M* = 2328.51 ms, *SD* = 2288.004 ms).

**Figure 5:**
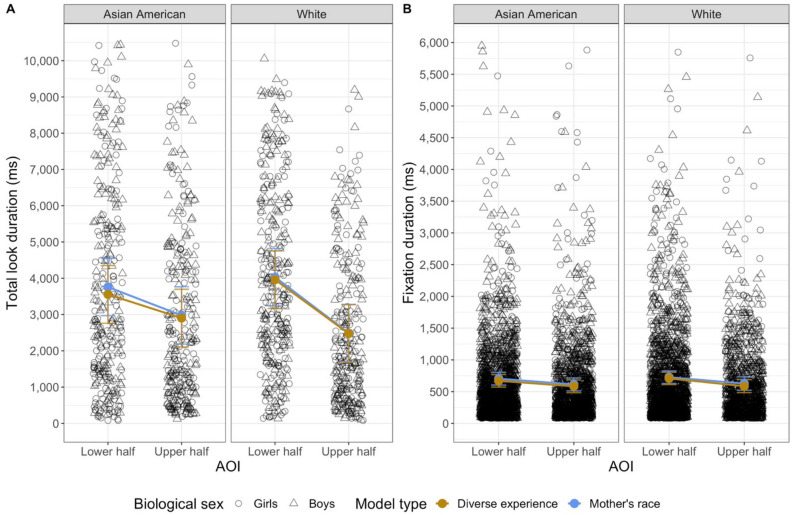
Duration of (**A**) total looking on each trial and (**B**) of individual fixations on each trial to the upper and lower regions, separated by Asian American and White faces. Each open circle is the duration on a single trial from a single infant (**A**) or a single fixation on a single trial from a single infant (**B**). The lines represent the estimated marginal means for each of the two models, and the error bars represent 95% Sidak-adjusted confidence intervals calculated from the models. Note, for clarity, the graph for the fixation durations does not include three fixations that were between 6000 and 8000 ms, but those fixations were included in the model. The biological sex of each infant is indicated by the shape of the point.

Infants’ looking varied as a function of the race of the stimulus. Both models that included *face race* as a fixed effect (the ones including mother’s race and diversity of face experience) revealed significant omnibus face race by AOI interactions: the model with mother’s race, *F*(1, 1011.69) = 7.63, *p* = 0.006, the model with diversity of experience, *F*(1, 1005.51) = 8.18, *p* = 0.004. As seen in Figure 5, infants looked longer at the lower halves of White faces (*M* = 3755.052 ms, *SD* = 2622.52 ms) than of Asian American faces (*M* = 3335.847 ms, *SD* = 2884.073 ms) and longer at the top halves of Asian American faces (*M* = 2578.705 ms, *SD* = 2456.024 ms) than of White faces (*M* = 2084.768 ms, *SD* = 2086.335 ms). Importantly, both of these effects were observed in both of the analyses that tested them.

**Figure 6:**
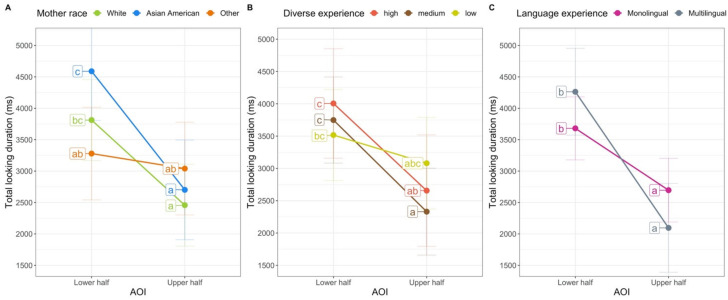
Looking at the upper and lower regions of the faces by (**A**) mother’s race, (**B**) diversity of experience, and (**C**) language experience, averaged across infants and trial type. The colored letters that correspond to each marginal mean point represent simple contrasts of the estimated marginal means with Tukey post-hoc corrected *p*-values for multiple comparisons. Marginal means with different corresponding letters (i.e., points “a” and “b” in panel c) significantly differ. Error bars represent 95% Sidak-adjusted confidence intervals.

Each model revealed a significant interaction between the experience variable (mother’s race, diversity of experience, or language experience) and AOI. These effects are presented in Figure 6. The model with mother’s race revealed an interaction between mother’s race group and AOI, *F*(2, 1117.32) = 11.11, *p* < 0.001. The estimated marginal means from the model are in the left panel of Figure 6, and the observed means are in Table 3. To probe the interaction further, we conducted simple comparisons among the estimated marginal means, using Tukey’s post-hoc corrected *p*-values (significant differences are indicated in Figure 6). Infants with Asian American mothers looked longer at the lower halves of faces than infants of non-White/non-Asian American mothers. In addition, infants of Asian American mothers and infants of White mothers looked longer at the lower halves of the faces than at the top halves of the faces; infants of non-White/non-Asian American faces did not look for different amounts of time to the two halves of the faces. The two groups of infants who saw stimuli that were the same race as their own mothers showed a bias to look at the lower half, whereas infants for whom all the stimulus faces were less familiar (with respect to their mother’s race) looked about equally to the two halves of the faces.

The model on the diversity of infants’ face experience revealed an interaction between diversity and AOI, *F*(2, 1026.45) = 5.65, *p* = 0.004. The estimated marginal means are in the middle panel of Figure 6. Although there were no differences in how long infants in the three groups looked at the upper or lower halves of the face, the post-hoc comparisons did reveal that only infants with high and medium diversity experience showed a significant preference for the lower half of the face; infants who had experience with only one race did not look for different durations to the upper and lower halves of the faces (see Figure 6).

Finally, the model with language experience revealed an interaction between language and AOI, *F*(2, 1009.29) = 14.96, *p* = 0.001. Although infants in the two groups did not have significantly different amounts of looking to the upper or lower regions, the interaction indicates that the difference between the looking to the upper and lower regions was greater for the multilingual infants. None of the models revealed any other significant effects or interactions.

### 3.3. Fixation-Level Analyses

Our *fixation-level analyses* examined infants’ eye gaze by evaluating *each individual fixation* in every trial. Specifically, we analyzed (1) the duration of each fixation, and (2) the location (i.e., upper or lower half) of each fixation. As for the trial-level analyses, we fit separate models for each experience variable as a fixed effect (mother’s race, diversity of race experience, and language experience), with the same fixed and random effects as described for the trial-level analyses described earlier. The models on the fixation-level data, however, also included a fixed effect of *fixation index*. This variable was unique for each fixation within a trial, and indicated the order of the fixations (e.g., an index of 1 was given to the first fixation, and index of 5 was given to the fifth fixation). This allowed us to assess how the observed associations between our experience variables change over subsequent fixations. We examined up to 3-way interactions between all variables except trial number and age in days. As described for the analyses of the total looking measure, we included a random intercept for the grouping variables participant and stimulus. Thus, each model was defined as follows (note that Stimulus Race was only included in the models including the experience variables mother’s race and race experience, and AOI was not included in the logistic regressions, in which the DV was 1 for a fixation to the lower region and 0 for a fixation to the lower region):

DV~(Experience variable + Stimulus race + Speech type + Index number + AOI ^3 + Trial number + Age in days + (... | Participant) + (... | Stimulus)

#### 3.3.1. Fixation Duration

**Figure 7:**
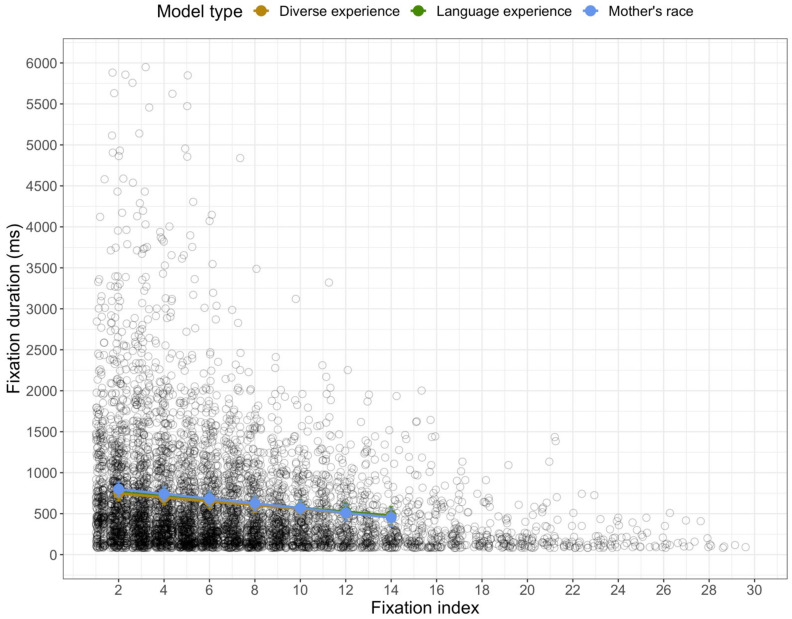
The duration of fixations as a function of fixation index. Each dot represents a single fixation from a single infant; the three lines represent the estimated marginal means from the three models. Note, this figure removed three fixations that had durations greater than 6000 ms (all fixation index <3), but all fixations were included in the model. In addition, as this figure demonstrates, the number of fixations decreased dramatically as the fixation index increased. In fact, 93% of the fixations were index 1 through 14. In future figures, we show model estimates to index 14. Error bars represent 95% Sidak-adjusted confidence intervals.

The first analyses were conducted on the duration of the individual fixations. Once again, all three models each revealed fixed effects of trial: the model with mother’s race, *F*(1, 6269.4) = 25.32, *p* < 0.001, the model with diversity of experience, *F*(1, 5753.3) = 27.94, *p* < 0.001, and the model with language experience, *F*(1, 5672.0) = 16.41, *p* < 0.001. Like the amount of looking to each AOI, the duration of fixations decreased as the session progressed. In addition, each of the models revealed a fixed effect of fixation index: the model with mother’s race, *F*(1, 6218.9) = 133.87, *p* < 0.001, the model with diversity of experience, *F*(1, 5706.3) = 127.75, *p* < 0.001, and the model with language experience, *F*(1, 5532.3) = 80.59, *p* < 0.001. Not only did fixation durations decrease over trials, they decreased over time within a trial (see Figure 7).

All of the models also revealed fixed effects of the AOI: the model with mother’s race, *F*(1, 6220.6) = 12.00, *p* < 0.001, the model with diversity of experience, *F*(1, 5721.5) = 14.15, *p* < 0.001, and the model with language experience, *F*(1, 5561.5) = 17.91, *p* < 0.001. In general, infants’ individual fixations to the lower region (*M* = 666.313 ms, *SD* = 705.47 ms) were longer than were their fixations to the upper region (*M* = 606.328 ms, *SD* = 657.948 ms) (see Figure 4).

There were also effects of stimulus characteristics on the duration of fixations. The models with mother’s race and diversity of face experience both revealed significant interactions of face race, speech type, and index number, model with mother’s race *F*(1, 6193.2) = 6.98, *p* = 0.008 and model with diversity of experience, *F*(1,5697.8) = 10.83, *p* = 0.001, and face race, AOI, and index number, model with mother’s race *F*(1, 6224.4) = 4.40, *p* = 0.036 and model with diversity of experience, *F*(1, 5697.8) = 10.83, *p* = 0.001. The model with diversity of face experience also revealed significant omnibus interactions of face race and speech type, *F*(1, 9.4) = 8.08, *p* = 0.018, face race and AOI, *F*(5681.5) = 5.55, *p* = 0.018, and speech type by index number, *F*(1, 5691.3) = 5.97, *p* = 0.015, but these omnibus 2-way interactions are subsumed by the omnibus 3-way interactions observed in both analyses. The analysis of language experience (that did not include face race as a variable) did not yield any significant effects or interactions related to stimulus characteristics.

**Figure 8:**
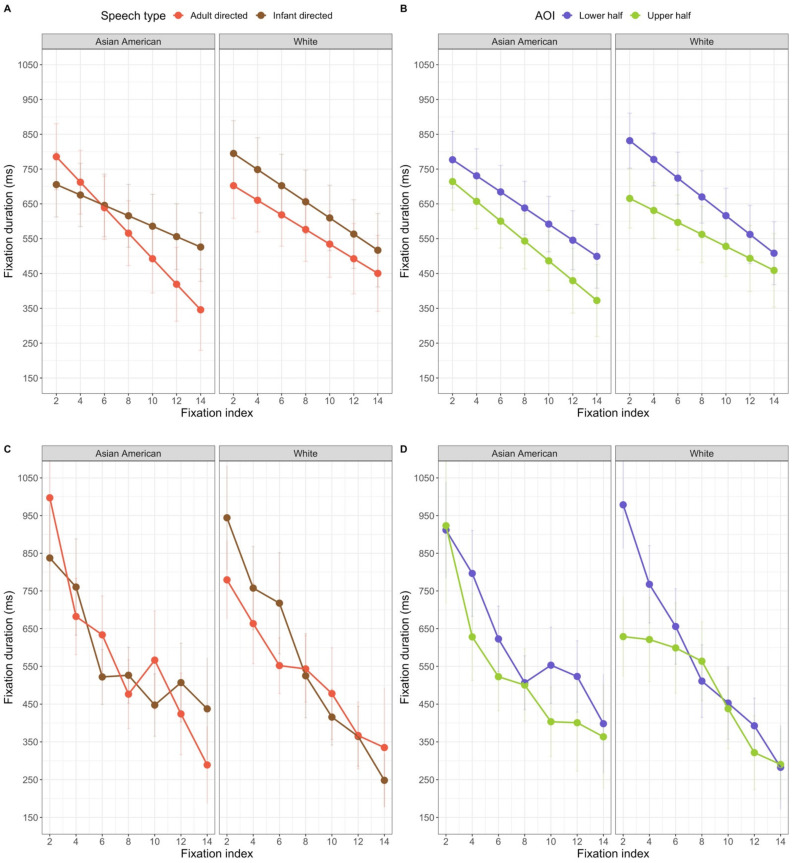
Top: The estimated marginal means for the duration of fixations as a function of (**A**) face race, speech type and fixation index and (**B**) face race, AOI, and fixation index (**B**), taken from the model including diversity of experience. Bottom: The observed means for the interactions of (**C**) face race, speech type, and fixation index, and (**D**) face race, AOI, and fixation index. Error bars represent non-adjusted 95% confidence intervals. Although fixations are only plotted to fixation index 14, all fixations were entered in the models.

The 3-way interaction between face race, speech type, and index from the model with diversity of experience is presented in the left half of Figure 8 (note that although in this and other figures we present only the first 14 fixation indices, all fixations were included in the models). It is clear that when viewing Asian American faces using adult-directed speech, the duration of infants’ fixations showed a more dramatic decrease than the duration of their fixations when viewing Asian American faces using infant-directed speech. When viewing White faces, infants consistently had longer fixations when the faces were using infant-directed speech. We compared the estimated means at indexes 2, 4, 6, 8, 10, 12 and 14, using a *False Discovery Rate* approach to correct for multiple comparisons. These comparisons did not yield any significant differences between the infant-directed and adult-directed stimuli for either race face at any index level (see Table 4). Thus, this interaction reflects qualitative differences in the change in the duration of infants’ fixation durations when viewing different stimuli.

The 3-way interaction between face race, AOI and index is presented in the right half of Figure 8. Post-hoc comparisons (*t*-tests) at the index level specified earlier (within each stimulus type), comparing the duration of fixations to the upper and lower regions revealed that fixations were longer to the lower region at every index level for the White faces (see Table 5). However, for the Asian American faces, the earliest fixations to the upper and lower regions did not differ significantly; the difference emerged as the trial progressed.

**Figure 9:**
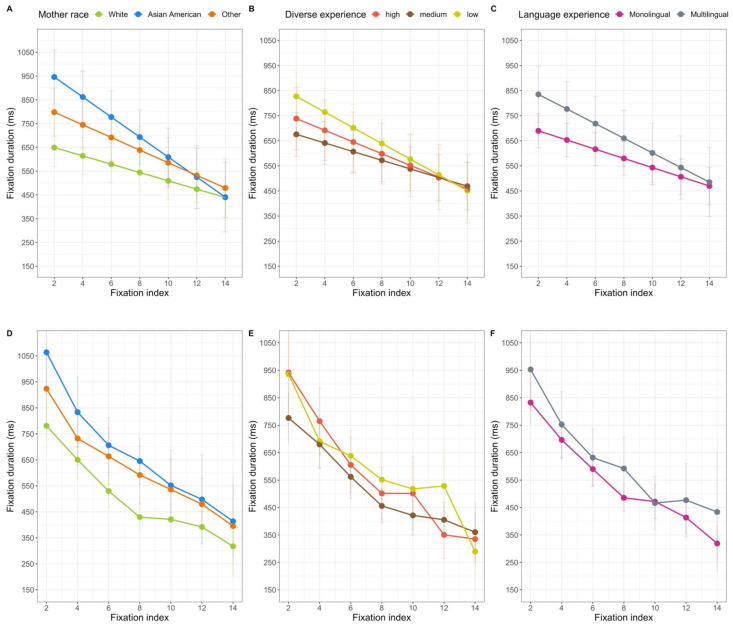
Top: The estimated marginal means for the duration of fixations at each fixation index as a function of experience: (**A**) mother’s race (**B**) diversity of experience, and (**C**) language experience. Bottom: the observed means for the duration of fixations at each fixation index as a function of experience: (**D**) mother’s race (**E**) diversity of experience, and (**F**) language experience. Error bars represent non-adjusted 95% confidence intervals. Although fixations are only plotted to fixation index 14, all fixations were entered in the models.

Finally, all of the models revealed effects of experience on infants’ fixation durations. There were significant effects of mothers’ race, *F*(2, 134.4) = 12.46, *p* < 0.001, diversity of experience, *F*(2, 113.2) = 3.72, *p* = 0.027, and language experience, *F*(1, 117.6) = 5.98, *p* = 0.016, on infants’ fixation durations in general. Each of these experience variables interacted with index number: the model with mother’s race, *F*(1, 6274.6) = 9.013, *p* < 0. 001, the model with diversity of experience, *F*(1, 5766.8) = 4.44, *p* = 0.012, and the model with language experience, *F*(1, 5700.6) = 4.22, *p* = 0.040. The estimated means for these interactions are in Figure 9. When looking at the effect of mother’s race, only three post-hoc comparisons revealed differences in the durations of fixations by the groups of infants; infants with Asian American mothers had longer fixations than infants with White mothers at index 2, *t*(111.57) = 4.46, *p* (adjusted) < 0.001, index 4, *t*(96.77) = 4.46, *p* (adjusted) = 0.004, and index 6, *t*(95.80) = 3.09 *p* (adjusted) = 0.032. In general, infants with Asian American mothers had the longer early fixations. The interaction with diversity of experience is in the middle panel of Figure 9. Post-hoc comparisons between the groups at each fixation index did not reveal any significant differences, all with a FDR-corrected *p* > 0.05. Inspection of the middle panel of Figure 9 suggests that infants with the least diverse experience showed the most dramatic decrease in the duration of fixations over the course of the trials; infants with medium and high levels of diversity showed a less extreme decrease in fixation duration as the trial progressed. Finally, the interaction with language experience is in the right panel of Figure 9. Again, none of the post-hoc comparisons between the groups showed significant differences at any of fixation indexes, all with a FDR-corrected *p* > 0.05. It appears that infants with more diverse language experience showed a stronger decrease in fixation duration than infants who primarily heard English. Although it is difficult to ascertain a general mechanism that can explain all of these effects, it is clear that infants’ fixation durations while viewing these stimuli were related to some aspect of their experience.

#### 3.3.2. Fixation Location

To examine whether infants’ fixations were more likely to be in the upper and lower halves of the face, we fit three logistic mixed-effects models with infants’ coded fixations on each trial as the dependent variable to the upper (coded as 0) and lower regions (coded as 1) of the faces. The models had the same variables as described earlier; however, we standardized (without centering) the continuous variables to aid in model convergence (i.e., Index number, Trial number, and Age in days).

**Figure 10:**
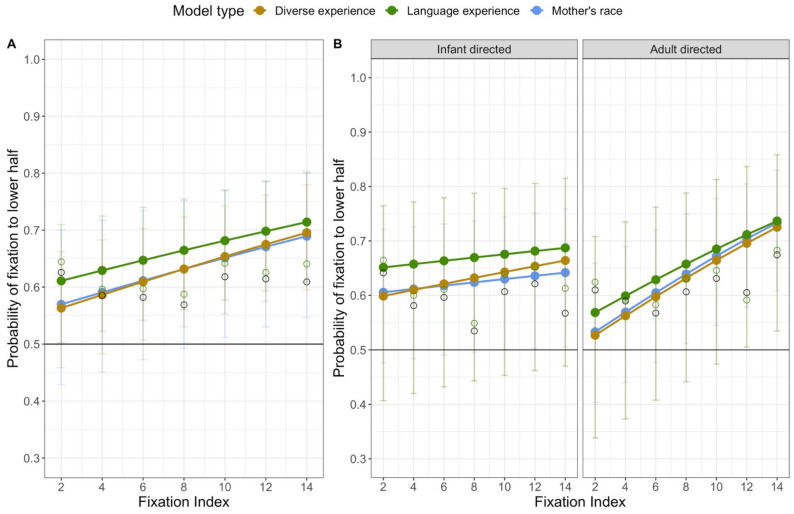
The probability of a fixation that is directed to the lower region of the face; higher numbers indicate a larger probability of fixations to the lower half: (**A**) depicts the fixed effect of Index, and shows how the probability of fixations to the lower half changes over fixation index. (**B**) depicts the interaction between speech type and fixation index and shows how the change in the probability of fixations directed to the lower half over fixation index is different for infant-directed and adult-directed stimuli. The line bisecting each figure at 0.50 indicates change (e.g., equal proportion of looking to the upper and lower halves). For each graph, the open circles represent the observed proportion of fixations directed to the lower half of the faces collapsed across all infants and for all trials; the green open circles only contain fixations from infants that were considered multilingual or monolingual and used for the language analyses, the black open circles represent fixations from all infants regardless of language experience. The filled circles connected by colored lines represent the marginal means from each of the models. The brown circles are the marginal means from the diversity of experience model, the green circles are from the language experience model, and the blue circles are from the mother’s race model. Error bars represent asymptotic upper and lower confidence limits.

Each model revealed a fixed effect of index: the model including mother’s race, *χ*^2^ (1, *N* = 98) = 20.48, *p* < 0.001, the model including diversity, *χ*^2^ (1, *N* = 89) = 33.24, *p* < 0.001, and the model including language, *χ*^2^ (1, *N* = 89) = 15.01, *p* < 0.001. The estimated number of fixations in the upper and lower region at each fixation index from all three models are presented in the center of Figure 10. Although at every level of the index variable there are more fixations to the lower half than to the upper half, the probability that a fixation is in the lower half increases across the trial.

The models also revealed stimulus effects on the probability that fixations were in the lower half. All three models revealed a significant interaction between speech type and fixation index: the model including mother’s race, *χ*^2^ (1, *N* = 98) = 11.29, *p* < 0.001, the model including diversity, *χ*^2^ (1, *N* = 89) = 9.37, *p* < 0.002, and the model including language, *χ*^2^ (1, *N* = 89) = 7.18, *p* = 0.007. As can be seen in the right panel of Figure 10, infants showed a relatively constant proportion of fixations to the lower region in the infant-directed stimuli, but a dramatic increase in the proportion of fixations to the lower region over the course of the adult-directed stimulus trials (recall that as the fixation index increases, the number of data points decrease, resulting in more variability in the data at the higher fixation indexes). Thus, as we observed in our other analyses, there are differences in the lower half bias for infant-directed and adult-directed stimuli.

**Figure 11:**
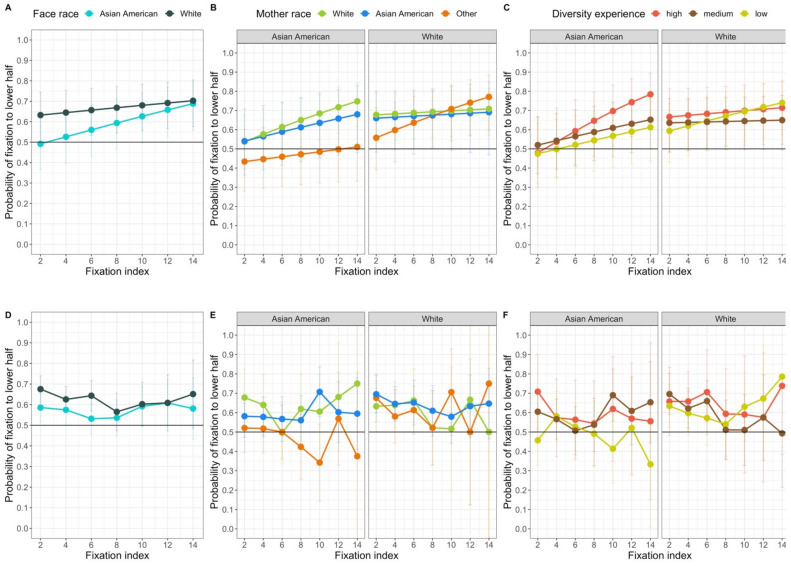
The probability of a fixation to the lower half of the face. Top: Model estimate for the probabilities on each index as a function of (**A**) face race, (**B**) face race and mother’s race, and (**C**) face race and diversity of experience. Bottom: Observed proportions on each index as a function of (**D**) face race, (**E**) face race and mother’s race, and (**F**) face race and diversity of experience. Error bars represent asymptotic upper and lower confidence limits. Although fixations are only plotted to fixation index 14, all fixations were entered in the models.

The mother’s race and diversity of experience models also revealed effects of face race. The model including the diversity variable revealed a significant interaction between face race and index number, *χ*^2^ (1, *N* = 89) = 6.95, *p* = 0.008, and a marginally significant interaction between face race, diversity of experience, and index number, *χ*^2^ (2, *N* = 89) = 5.87, *p* = 0.053. The model including mother’s race revealed a significant interaction between face race, mother’s race, and index number, *χ*^2^ (2, *N* = 98) = 11.34, *p* = 0.003. As illustrated by the right half of Figure 11, in general, the likelihood that early and late fixations are in the lower half was different for the two face races. When viewing White faces, infants’ fixations were likely to be in the lower region from the first fixations. When viewing Asian American faces, in contrast, the early fixations are equally likely to be in the upper or lower region, and fixations become increasingly likely to be in the lower region as the trial progresses. Moreover, this effect varies as a function of infants’ everyday experience with different races, as evident by the significant interaction with mother’s race and marginally significant interaction with diversity of face experience.

The left panel of Figure 11 shows how this interaction of face race and index varies as a function of mother’s race. In general, all three groups of infants responded differently to Asian American and White faces, but they did so in different ways. Interestingly, although infants with Asian American and White mothers showed the general pattern for Asian American and White faces, infants whose mothers are neither Asian American nor White showed a different pattern. These infants were approximately equally likely to look at the upper and lower regions of the Asian American faces, and showed an increase in their looking to the lower region of the White faces. A clearer effect of experience emerges when looking at the means for the interaction with diversity. It is clear that how much infants treat the Asian American and White faces differently depends on the diversity of their experience. Infants with highly diverse experience show a dramatic increase in the proportion of their looks to the lower half of Asian American faces, and a consistent amount of looking to the lower halves of White faces. Infants with low diversity in their face experience, in contrast, show a gradually increasing proportion of looking to the lower halves of Asian American and White faces.

### 3.4. Time Course Analyses

Our final analyses were on how infants’ gaze position changed over time. Using a procedure described in Beckner et al. [29], we conducted a time course analysis to examine how infants’ bias for the lower half of the face emerges and changes as the trial unfolds. It would be useful to conduct these analyses as a follow up to all the effects that emerged from the previously described analyses, but the design of this experiment did not provide sufficient power to conduct all those analyses. Specifically, the permutation approach described by Beckner et al. involves conducting uncorrected *t*-tests comparing the preference for one region (the lower region in the present case) to chance, and evaluating whether clusters of these uncorrected *t*-tests are different from that expected by chance as determined by permutation analyses. Given the sample rate of our eye tracker, in our 7500 ms trials, we should have 900 samples for each subject on each trial, with each sample reflecting whether they were looking at the upper half of the face, the lower half of the face, or neither for each time sample on any given trial.

Not every infant had data at every sample on every trial. These missing data create a signal-to-noise ratio problem that is resolved by averaging across a large number of trials to derive a subject-weighted average for each time sample. However, in the present study, infants were only presented with eight total trials, and a maximum of two trials of each stimulus type. As a result, the signal-to-noise ratio was only partly solved by creating the subject-weighted averages. We improved the signal-to-noise ratio further by calculating average scores across multiple time samples, rather than by examining how infants’ looking changed in each sample. We created bins of 250 ms and determined the proportion of samples in each time bin that were directed to the lower region. This approach increased the probability that infants provided usable data for each of our time bins on a given trial, providing more power to conduct our time course analyses with the small number of trials included in this study.

Averaging across time bins allowed us to conduct our analysis on fewer trials, but it did not allow sufficient power to test our between-subjects manipulations. Subdividing our sample to evaluate how mother’s race, diversity of face experience, or language experience influenced the time course of infants looking behavior significantly reduced the number of infants that provided usable data for each time bin, even after we averaged across multiple time bins. This is a known limitation of this approach [52].

Here we analyzed the time course data for our within-subject manipulations (e.g., speech type and face race), collapsing across our between-subject variables. This provided us with the maximum number of participants for each time bin and allowed us to robustly assess moment-to-moment changes in looking behavior. Specifically, we conducted two time course analyses: (1) the time course collapsed across all trial types and (2) the time course for each of the four stimulus types (Asian American/infant directed, Asian American/adult directed, White/infant directed, White/adult directed). We had sufficient power to conduct these two analyses and these analyses alone illustrate what a time course analytic approach adds to the overall understanding of infants’ viewing of these stimuli. In addition, by examining the patterns that emerged across the time course analysis for the four separate trial types we can further understand the effects of stimulus type observed for looking duration, fixation duration, and fixation location.

As a first step, we created time bins of 30 raw time samples (each 8.33 ms), or 250 ms. For each of these time bins, we calculated the proportion of samples in which we observed a look to the upper or lower region that were devoted to the lower region (note that we are examining infants’ looking on each *sample*; individual fixations occur over many samples, and therefore changes over these 250 ms bins do not reflect the same changes as observed across fixations within a trial). For example, if on a particular bin, an infant allocated gaze to the lower half of the face on 12 samples and allocated gaze to the upper half of the face on 18 samples, the proportion score for that time bin would be 0.40. We calculated the proportion for each 250 ms time bin for the first 7500 ms of each trial; because the trials were different length, we analyzed only the first 7500 ms of all the trials. This yielded a lower half preference score for 30 time bins on each trial for each infant.

We used the proportion scores in each time bin for each trial to calculate several subject-weighted means. First, for each infant, we created an average time course for each infant by averaging the lower region proportion across all trials, regardless of trial type. We then averaged these individual subject time courses to create a subject-weighted time course collapsed across all the four trial types. We also created for each infant separate time courses for each trial type by averaging the proportion scores on each time bin across trials of the same type. We used these trial type time courses to create separate subject-weighted time courses for each trial type.

For each time course we conducted a permutation analysis to determine when during the trial the infants’ bias to the lower half of the face was significantly different from chance. Specifically, we generated a null distribution for comparing clusters to chance by running the permutations 1000 times. For each permutation, we derived *cluster masses* by identifying clusters of consecutively significant uncorrected *t*-tests and summing the *t*-statistics for these clusters. The absolute value for the largest cluster mass was stored in our null distribution on each iteration of the permutation, yielding a distribution of cluster masses that we would expect from random permutations of the data (i.e., chance). We then compared the cluster masses in our observed data to the distribution of the largest cluster masses in our permutations. If the cluster masses in the observed data were larger than 95% of the cluster masses in our null distribution, then these observed clusters were significantly larger than expected by chance. For our analysis collapsed across all four trial types, 95% of our cluster masses in our null distribution had a summed *t*-value of less than 9.60. For our separate analyses for each trial type, each permutation yielded a different threshold of significance, but for all of them at least 95% percent of our cluster masses had summed *t*-values of less than 7.76. Thus, a cluster mass exceeding these values for each respective analysis would indicate a cluster that is significantly different from chance as indicated by our permutations.

**Figure 12:**
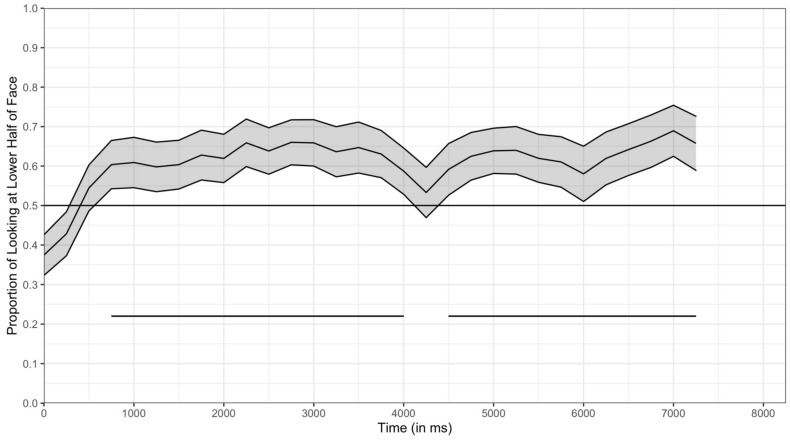
The significant clusters of *t*-tests comparing the proportion of fixations to the upper region to chance (0.50) are indicated by a line at the bottom of the figure and are listed in Table 5. Recall that the significance of these clusters was established using a permutation analysis which therefore helps to control for multiple comparisons. It can be seen that overall infants fixated on the lower region more than chance between 750 and 4000 ms and between 4500 and 7250 ms, so for nearly the entire trial.

The results of the time course collapsed across trial types is presented in Figure 12. This figure represents the proportion of looking in each 250 ms bin that was directed toward the lower half of the face. Chance is 0.50 and represents an equal proportion of looking at the two halves of the face. When the line is above 0.50, there was more looking in the lower half and when the line is below 0.50 there was more looking directed towards the upper half. It is clear that in the first 500 ms, infants are slightly more likely to fixate on the upper half of the face than the lower half, but by 1000 ms into the trial were more likely to look toward the lower half of the face (i.e., above 0.50 in the figure; see Table 6).

**Figure 13:**
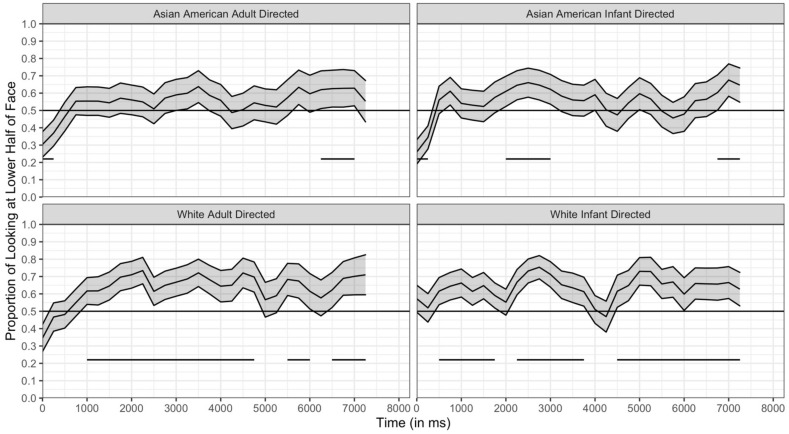
Subject-weighted mean time course for each trial type. The *x* axis represents time bins and the *y* axis represents the proportion of looking at the lower half of the face. Each line represents the subject-weighted mean proportion of looking at the lower half of the face and the shading around the lines represents 95 percent confidence intervals. The horizontal bar bisecting the *y* axis represents chance (0.50). Individual line segments displayed below the subject-weighted mean time course for each condition indicate clusters that were significantly different from chance as indicated by our permutation analysis.

The time courses for four different trial types are presented in Figure 13, and the significant clusters are in Table 7. In each trial, infants showed a statistically significant preference for the lower half of the face, but at different points in the trial for each type of stimulus. Specifically, when viewing White faces, infants showed significant preferences for the lower region early in the trial—for both adult-directed and infant-directed speech, the proportion of infants’ looking to the lower region was significantly greater than chance by 1000 ms. In addition, when viewing these stimuli, infants showed sustained preference for the lower region for much of the trial. When viewing Asian American faces, infants did show a bias for the lower region, but it occurred later for both stimuli, and was only beginning to emerge at the end of the 7500 ms of the time course analysis in the adult-directed stimuli. In general, these conclusions are consistent with the analysis presented earlier in which infants’ total amount of looking to the lower regions were greater when infants were viewing the White faces than when looking at the Asian American faces, and with the analyses of the location of each fixation at each index value.

The time course analyses also reveal insight into the effect of the speech type on infants’ lower half preferences. For both Asian American and White faces, a lower half bias emerged earlier in the trial when the woman was using infant-directed speech (the left column of figures in Figure 13) than when she was using adult-directed speech (the right column). Recall that these two sets of stimuli were the same woman speaking. Thus, infants preferred the lower half earlier when the same woman was speaking in infant-directed speech than when she was speaking in adult-directed speech. Again, it must be remembered that infants had only the visual information, and these differences did not reflect the influence of the auditory differences. In general, these analyses, therefore, confirmed the effects observed from the analyses from the total looking durations and the fixations and the location of each fixation.

However, the time course analyses provide finer grained understanding than was revealed from the other analyses reported here. As can be seen in Figure 13, infants initially showed a significant bias towards the upper half of the face for both adult-directed and infant-directed Asian American faces. Thus, not only do these analyses provide insight into when during the trial the lower half bias emerged, they also show that when looking at Asian American faces, infants actually shift from an upper half bias to a lower half bias over the 7500 ms of the trial. This bias to initially look at the upper halves of the Asian American faces may help to explain why overall the lower half bias was weaker for these stimuli than for the White stimuli.

## 4. Discussion

In this study, we explored infants’ eye gaze as they viewed dynamic faces. Two aspects of our work inform our conclusions. First, we did not report a single set of analyses conducted on an a priori selected single DV. Rather, our conclusions are based on identifying the most consistent, robust results across several different exploratory analyses conducted on several different DVs, much as is done in the multiverse analytic approach [32,33]. In the present experiment, we analyzed infants’ looking at different time scales and evaluated those analyses for consistent effects. The second aspect of our work that informed our conclusions is that we did not index only one aspect of the infants’ experience. Instead, we evaluated infants’ gaze patterns as they inspected our dynamic stimuli as a function of their experience with different races and their language experience. Thus, we were able to assess how both kinds of experience contributed to differences in looking in the same sample of infants.

Several patterns were evident in the data. First, as is often the case in studies with infants, the longest periods of looking were early in the session and early in the trial. Even in this short experimental session, infants’ looking duration significantly decreased over time. The models for duration on each trial and the duration of each fixation revealed significant effects of *trial*, due to infants’ overall amount of looking and the duration of their fixations being longer on the initial trials than on the later trials. In addition, the duration of infants’ fixations decreased within a trial, with the longest fixations occurring early in the trial (i.e., with low index numbers) and shorter fixations occurring later in the trial (i.e., with high index numbers).

A second consistent finding was that infants preferred the lower regions of the faces, as has been observed in other studies with infants in this age range using dynamic stimuli (e.g., [12]). This was observed in our analyses of the total amount of looking to the lower or upper halves of the faces on each trial, the duration of fixations to the lower or upper halves of the faces, and the time course analyses evaluating the proportion of infants’ looking on each 250 ms time bin during the trials. Moreover, we observed this effect in each of the models, regardless of how we defined experience (and as a result which subset of infants were included in those models). Thus, this bias for the lower regions of the faces in this study is robust and is not the result of which subset of data we analyzed or which dependent measure we selected.

This lower region bias depended on the stimuli infants were viewing. Infants showed different biases for Asian American and White faces, consistent with research with older children and adults [10,11,53]. However, previous studies have shown that both Chinese adults [10] and European adults [11] look more at the eyes of White faces and the mouth regions of Asian faces, whereas here we observed infants had a larger preference for the lower half of the White faces. This reversed bias might reflect something about our Northern California infants’ daily face experience (see [25]). It is more likely that the difference between our results and those reported previously are due to differences in stimuli and methodology. For example, Krasotkina et al. [19] reported that White infants showed a stronger preference for eyes relative to mouths of own-race White faces than to other-race Asian faces when shown a series of static images over many habituation trials that each could be up to 40 s in duration. Similarly, Lee and colleagues found that White infants showed biases for the eyes/upper region of own-race faces and biases for mouth/lower region of other-race faces when they viewed 30 s videos of a woman counting with a neutral face [20,21]. Thus, one possibility is that a bias to look at the upper region of White faces and lower region of Asian American faces emerges over a longer period than in our 7.5 to 10 s trials. However, Wilcox et al. [6] found that a sample of predominantly White infants looked more at the eyes of a White woman talking in 4 s trials. In addition, using relatively long stimuli (30 s videos), Smith and colleagues [18] found that infants looked at the eyes of women talking. It is therefore not clear that our results differed from others because of the duration of our trials. Nevertheless, our time course analysis (Figure 13) suggests that a bias for the lower half may have been emerging late in the trials with Asian American faces. Note that our analyses of infants’ overall looking conflict with previous findings, but our analyses examining changes in infants’ looking across the course of the individual trials provided insight into the source of this discrepancy. This is a question that should be addressed in future research.

Our stimuli may have induced more looking to the lower region because they engaged language-related processing. Biases for the mouth have been tied to infants’ developing language abilities [12,14], even when looking at non-linguistic stimuli [7]. In our study, infants’ bias to look at the lower half of the face was related to whether the woman was using infant- or adult-directed speech. Specifically, when the woman was using infant-directed speech, our infants were more likely to fixate on the lower region of the face and maintained this preference throughout the trial, perhaps reflecting that these stimuli induced more language processing. Given that the auditory information was incongruent with the visual information in the stimuli (i.e., we removed the speech information and replaced it with music), infants may have spent more time looking at the lower region of the face because they were trying to understand what the woman was saying, similar to when infants are viewing a woman speaking an unfamiliar language [14,15]. It is worth noting that infants’ do discriminate the visual features of infant- and adult-directed speech [38], and thus our effects likely reflect infants’ sensitivity to the head and facial movements associated with infant-directed speech [54]. It is also possible that the infant-directed stimuli were simply more positive [55], which induced different scanning. The point is that although we do not know precisely why infants showed different preferences as a function of the stimulus characteristics, our results robustly reveal that infants’ bias to look at the lower region of the face varies for different types of faces and stimuli.

These preferences were moderated by the infants’ own experience. In this study, we used three independent indices of experience, each capturing a different aspect of infants’ daily life that may influence their face processing. Interestingly, each of our experience variables—mother’s race, diversity of race experience, and language experience—yielded significant interactions with AOI in multiple DVs. In terms of mother’s race, we found that infants of Asian American mothers (1) had longer looking to the lower regions than infants of non-Asian/non-White mothers, (2) had longer individual fixations than infants of White mothers, and (3) showed the biggest difference in fixating on lower and upper regions. In contrast, infants whose mothers were non-Asian/non-White looked equally to the upper and lower regions and showed differences in their lower region bias as a function of the race of the face. Although these results do not replicate precisely the effects reported in the literature for Asian and White infants’ processing of own and other race faces, we observed a consistent pattern of the effect of mother’s race on how infants look at faces across our DVs.

Clearer results were obtained for the effect of the diversity of race experience, extending previous findings about differences in face scanning by White infants from more or less racially diverse communities [25]. Infants in our sample with the greatest amount of diversity in their daily face experience showed the biggest difference in their attention to the upper and lower half of the faces as revealed by their duration of looking in the trial and the probability of a fixation to the lower region. Moreover, there was a hint of an interaction between diversity of experience and face race; infants with the most diverse experience showed the most dramatic differences in terms of the location of their looks to the upper and lower halves of the Asian American and White faces. However, this was observed in only one analysis with only one of our DVs, thus this conclusion is speculative and must be replicated before strong conclusions can be drawn.

Finally, we obtained modest support for the effect of language experience consistent with findings that bilingual infants have stronger preference for mouths [7,14]. In our sample, multilingual infants had a larger difference in the duration of looking to the upper and lower regions of the faces. In addition, multilingual infants had longer individual fixations than monolingual infants (regardless of AOI), indicating differences in their face processing. Taken together, these findings indicate that infants’ looking at faces is related to aspects of daily experience. When considering this collection of effects, it is important to keep in mind that there was little overlap in these groupings, so these experience variables are not simply different labels of essentially the same groups (see Table 1).

It should be pointed out that we did not observe any effect of experience in our ANOVAs on aggregate scores. There are several possible reasons why the linear mixed-effects models uncovered the contribution of experience whereas the ANOVA did not. One possibility is that the effect is not robust and strong conclusions should not be drawn. However, because all of our trial-level and fixation-level analyses yielded effects of experience, it does appear that these effects are robust. A second possibility is that the ANOVA approach was not sensitive to the effect of experience. Because the linear mixed-effects models controlled for variation due to individual subjects and specific stimuli—as well as making it possible for us to include all of our subjects in our analyses—they may have been more sensitive to these effects than the ANOVA on the aggregate scores. Finally, the ANOVAs were conducted on preference scores, whereas the linear mixed-effects models were conducted on duration of looking or location of a fixation. Thus, our different DVs may be differentially sensitive as indices of experience on infants’ gaze in this task. 

It is important to point out that our results show the strongest effects for diversity in experience on infants’ eye gaze patterns. Very little work has examined differences in how infants scan faces as a function of the diversity of their experience. The little work that has addressed this has shown that infants from more diverse communities or biracial infants scan faces differently than infants with less diverse communities or monoracial infants [25,26]. The present findings are consistent with these previous findings, and further suggest that differences in experience with faces induce differences in how infants learn to look at faces.

In general, the results of this study contribute to a body of literature indicating that over development, infants learn how to look as a result of their experience. Moreover, infants’ looking is important to their learning. Findings that infants in the second half of the first year look more at the mouths of talking faces, especially when the language task is more challenging (e.g., the face is speaking an unfamiliar language [14]) are consistent with the conclusion that infants look to the mouth region to disambiguate these contexts. The stimuli in the present study may have been particularly challenging for infants in the early stages of language acquisition. Recall that in our stimuli, the speech stream was removed and infants heard music as they viewed the talking faces. Thus, infants had visual cues that the woman was talking, but those visual cues conflicted with the auditory information.

Our conclusions are based on the pattern of results obtained from many different analyses. Rather than selecting a single dependent variable and a single analytic approach, here we examined the patterns we obtained across the analysis of several different dependent variables and when analyzing experience in different ways. Thus, we have confidence that our primary conclusions are robust and are not the function of decisions we made selecting a single dependent variable or characterizing experience in just one way. Because of parents failing to completely report some of our experience variables, we obtained consistent findings even when we analyzed different subsamples. Thus, like the recent work using a multiverse analysis approach [32,33], we reduce the possibility that our conclusions are based on decisions we made about how to analyze our data.

Note that our results also suggest that *multiple aspects of experience* contribute to infants’ scanning of faces. Although our analyses show a robust and consistent effect of experience, one consequence of the data analytic approach we adopted is that we are unable to clearly differentiate between different kinds of experience. Specifically, we did not have sufficient power to include all of these variables in a single analysis. However, these different ways of capturing experience did yield different ways of dividing up the sample. For example, as seen in Table 1, there were similar numbers of infants who were classified as high, medium or low diversity in the multilingual and monolingual groups. Additionally, there were multilingual and monolingual infants in each of the mother’s race groups, although a higher proportion of infants with White mothers were monolingual than the other groups. Mother’s race was not simply a proxy for how diverse the infants’ experience was with respect to race—the proportions of infants who were classified as having high, medium, or low diversity were similar for each mother’s race group. Thus, taken altogether, the results suggest that experience is related to the bias infants have to look at the lower regions of the face, not that a specific type of experience is required. It is possible that during the second half of the first year, infants learn to look at the lower regions of faces for many reasons, and the effects we observed reflect the influence of several factors on infants’ looking behavior. Moreover, it is possible that these two types of experience are actually part of a single system. As infants become more attuned to language, and begin to look at the mouths of people who are speaking, they may learn more about the relative importance of the eye and mouth regions of faces to learn about faces and language. This learning may be influenced by the particular faces they encounter, and infants who encounter more diverse faces have a different training set than do infants who encounter less diverse faces. This, of course, is speculative, but it illustrates how selectively measuring some aspects of experience but not others can result in an incomplete understanding of development.

In summary, the results reported here are generally consistent with others reported in the literature. Infants between 7 ½ and 10 ½ months of age are biased to look at the mouths of faces, and these biases are stronger for some face stimuli than others. In addition, the strength of this bias is related to the infants’ language and face experience. Unlike previous studies, the present conclusions were not a function of the particular dependent measure, specific analysis, or way of characterizing experience. Rather, this study shows that by reporting the results of many different exploratory analyses and focusing on the converging findings, we can have confidence that our conclusions are based on robust effects that were less likely to be the result of specific experimenter decisions.

## Figures and Tables

**Table 1:** Number of infants in each experience group.

Diversity of Experience	Mother’s Race			Total
	White	Asian American	Non-White/Non-Asian American	
Low	16	5	10	31
	(14/0/2)	(2/3/0)	(5/3/2)	(21/6/4)
Medium	21	8	10	39
	(18/2/1)	(4/4/0)	(6/3/1)	(28/9/2)
High	9	5	5	19
	(6/3/0)	(4/1/0)	(4/1/0)	(14/5/0)
Not reported	2	4	3	9
	(1/1/0)	(1/1/2)	(1/1/1)	(3/3/3)
**Total**	48	22	28	98
	(39/6/3)	(11/9/2)	(16/8/4)	

*Note*. In each cell of the table, the numbers in parentheses indicate the classification for infant’s language experience. Infants were classified as monolingual English (first number reported), multilingual (middle number reported), and monolingual in a language other than English or language experience was not reported (third number reported). Thus, in the upper left hand cell, there were 16 infants who had White mothers and low diversity of experience; 14 of these infants were English monolingual, 0 were multilingual, and 2 had some other language experience.

**Table 2:** Mean looking on each trial (SD) in ms to the lower and upper halves of the faces averaged across infants.

Trial	Half	
	Lower	Upper
1	4811.686 (2598.340)	2686.030 (2380.155)
2	3868.754 (2695.709)	2832.908 (2198.232)
3	4017.629 (2677.006)	2794.843 (2274.731)
4	3829.146 (2611.052)	2745.624 (2210.318)
5	3625.424 (2661.710)	2878.474 (2503.786)
6	3588.600 (2608.935)	2767.485 (2259.237)
7	3746.645 (2663.634)	2524.889 (2128.873)
8	3609.464 (2507.024)	2438.688 (2025.315)

**Table 3:** Observed mean (SD) looking time for the upper and lower halves of the faces for each experience group, as reflected in the 2-way interactions between experience and AOI in Figure 6.

Experience Variable	Group	*N*	Half	
			Lower Half	Upper Half
Mother’s Race	Asian American	22	4270.123 (2965.359)	2370.069 (2466.879)
	White	48	3601.218 (2695.414)	2129.799 (2119.374)
	Other	28	2880.059 (2542.746)	2620.941(2381.717)
Diversity of Experience	Low	31	3230.321 (2579.653)	2719.295 (2544.355)
	Medium	39	3509.517 (2708.892)	2009.844 (1931.018)
	High	19	3606.804 (2878.657)	2277.076 (2452.206)
Language Experience	Monolingual	66	3415.661 (2707.057)	2293.330 (2232.968)
	Multilingual	23	4069.642 (2779.548)	1849.599 (1969.672)

**Table 4:** The contrasts between fixation duration to the infant-directed and adult-directed stimuli as a function of face race, following up the 3-way interaction of speech type by face race by index from the diversity model. The *Estimate* is the estimated difference between the means from the model and the *SE* is the standard error of those estimates. The *p*-values were adjusted using the False Discovery Rate method.

Face Race	Index	Estimate	SE	*t*	df	*p*	*p* (Adjusted)
Asian American	2	−80.064	50.014	1.600	6.562	0.156	0.274
	4	−36.71	46.373	0.792	4.841	0.466	0.501
	6	6.645	45.152	0.147	4.338	0.890	0.890
	8	49.999	46.542	1.074	4.88	0.333	0.388
	10	93.353	50.326	1.855	6.647	0.108	0.274
	12	136.708	56.022	2.44	10.168	0.034	0.241
	14	180.061	63.114	2.853	16.311	0.011	0.159
White	2	92.633	50.036	1.851	6.578	0.109	0.274
	4	88.264	46.341	1.904	4.837	0.117	0.274
	6	83.895	44.926	1.867	4.263	0.131	0.274
	8	79.526	46.004	1.729	4.671	0.149	0.274
	10	75.157	49.41	1.521	6.189	0.178	0.276
	12	70.788	54.711	1.294	9.261	0.227	0.318
	14	66.419	61.419	1.081	14.631	0.297	0.378

**Table 5:** The contrasts between fixation duration to the upper and lower halves of faces as a function of face race, following up the 3-way interaction of AOI by face race by index from the diversity model; significant effects indicate that the fixations were longer to the upper region than the lower region at that index value. The *Estimate* is the estimated difference between the means from the model and the *SE* is the standard error of those estimates. The *p*-values were adjusted using the False Discovery Rate method.

Face Race	Index	Estimate	SE	*t*	df	*p*	*p* (Adjusted)
Asian American	2	62.752	34.587	1.814	5604.956	0.070	0.081
	4	73.417	29.264	2.509	5410.858	0.012	0.015
	6	84.082	27.157	3.096	5244.996	0.002	0.004
	8	94.748	28.976	3.27	5337.407	0.001	0.003
	10	105.413	34.097	3.091	5535.014	0.002	0.004
	12	116.079	41.312	2.809	5656.304	0.005	0.008
	14	126.744	49.715	2.549	5707.990	0.011	0.015
White	2	166.12	35.39	4.695	5730.656	<0.001	<0.001
	4	146.65	29.659	4.944	5670.677	<0.001	<0.001
	6	127.177	27.212	4.673	5623.512	<0.001	<0.001
	8	107.704	28.889	3.728	5677.153	<0.001	<0.001
	10	88.231	34.087	2.588	5741.784	0.010	0.015
	12	68.759	41.503	1.657	5760.326	0.098	0.105
	14	49.286	50.162	0.982	5756.749	0.326	0.326

**Table 6:** Significant clusters for chance comparison permutation analysis collapsed across all four of the permutations. The threshold of significance for our cluster masses as determined by the permutation analyses was 9.60.

Cluster Index	Sum *t*-Statistic	Onset	Offset	*p*-Value
1	57.9316	750	4000	0.000
2	48.9860	4500	7250	0.000

**Table 7:** Significant clusters for each chance comparison permutation analysis. Our threshold of significance was established by our permutation analyses; at least 95% of the permutations for each trial type yielded maximum cluster masses that were less than 7.76.

Face Race	Speech Type	Cluster Index	Sum *t*-Statistic	Onset	Offset	*p*-Value
Asian American	Infant Directed	1	11.3910	0	250	0.019
		2	15.9730	2000	3500	0.009
		3	8.7229	6750	7250	0.042
	Adult Directed	1	8.7767	0	500	0.047
		2	9.4549	6750	7750	0.037
White	Infant Directed	1	19.7795	500	1750	0.008
		2	34.2318	2250	3750	0.000
		3	43.4009	4500	7250	0.000
	Adult Directed	1	67.2777	1000	4750	0.000
		2	9.7633	5500	6000	0.035
		3	13.7549	6500	7250	0.015

## Data Availability

The data presented in this study are available in Supplementary Materials.

## Supplementary Materials

The following are available online at https://osf.io/anrgv/.
